# Why do older adults stand-up differently to young adults?: investigation of compensatory movement strategies in sit-to-walk

**DOI:** 10.1038/s41514-022-00094-x

**Published:** 2022-09-05

**Authors:** Eline van der Kruk, Paul Strutton, Louis J. Koizia, Michael Fertleman, Peter Reilly, Anthony M. J. Bull

**Affiliations:** 1 Biomechatronics & Human-Machine control, Department of Biomechanical Engineering, Faculty of Mechanical Engineering (3me), Delft University of Technology, Delft, the Netherlands; 2 Musculoskeletal Mechanics group, Department of Bioengineering, Faculty of Engineering, Imperial College London, London, UK; 3 The Nick Davey lab, Department of Surgery & Cancer, Faculty of Medicine, Imperial College London, London, UK; 4 Cutrale Perioperative and Ageing Group, Department of Bioengineering, Faculty of Engineering, Imperial College London, London, UK

**Keywords:** Motor control, Ageing, Signs and symptoms, Neural ageing

## Abstract

Functional motor redundancy enables humans to move with distinct muscle activation patterns while achieving a similar outcome. Since humans select similar strategies, there seems to be an optimal control. However, older adults move differently to young adults. The question is whether this is this due to an altered reinforcement scheme, altered sensory inputs, or due to alterations in the neuromusculoskeletal systems, so that it is no longer optimal or possible to execute the same movement strategies. The aim of this study was to analyse natural compensation strategies in the vital daily-life-task, sit-to-walk, in relation to neuromuscular capacity and movement objectives in younger (27.2 ± 4.6 years, *N* = 27, 14♀) and elderly (75.9 ± 6.3 years, *N* = 23, 12♀) adults. Aspects of the neuromuscular system that are prone to age-related decline and feasible to quantify were assessed (i.e. strength, nerve conductivity, fear of falling). Kinematics and muscle activity were recorded and joint kinetics were estimated using biomechanical models. Elderly men consistently used their arms when standing up. This strategy was not associated with a lack of or a reduction in strength, but with a reduction, but no lack of, ankle joint range of motion, and with increased fear of falling. The results show that humans preferentially maintain a minimum threshold of neuromuscular reserve to cope with uncertainties which results in compensation prior to coming up against physical limitations. Smaller base of support while standing up, a compensatory strategy with possibly greater risk of falls, was associated with muscular weakness, and longer nerve conduction latencies.

## Introduction

Over the course of millions of years, the human body and the way we propel ourselves has evolved into what we now consider regular daily life activities such as walking, running, or standing up. The human anatomy has over 200 joints and 600 muscles and can therefore complete the same movement task in many ways and at wide ranges of muscular co-contraction levels. This is called functional redundancy. Theoretically, each person could therefore move with distinct muscle recruitments, yet we all seem to move with stereotypical movement patterns^1^.

Within this functional redundancy, the fundamental idea has emerged that humans select the best recruitment to achieve a task. The basal ganglia control command centres in the brain stem based on choice, motivation and context, which activate specific motor control programs. The coordination is adapted to external events through sensory input. Within the redundancy of neuromuscular systems, a motor pattern can be rewarded by external rewards or perception of success, which leads to reinforcement of these motor patterns. Mathematically, this is equivalent to the process of optimizing (minimizing) a cost function associated with the movement, known as the optimal control principle. Yet, as humans age, their movements change; elderly adults move differently to young adults. The question is whether this is this due to an altered reinforcement scheme, altered sensory inputs, or due to alteration in the neuromusculoskeletal systems, so that it is no longer optimal or possible to execute the same movement strategies.

The rate and onset of age-related physiological decline differs between individuals and between components of the neuromuscular systems. Decline of neuromusculoskeletal capacity generally starts at a relatively young age of around 20 years^2^. Since the human movement system has physiological redundancy (reserve), initially no movement limitations arise due to early physical decay. However, over the course of several years, humans do adapt their movements. Adaptation, or compensation, is achieved via altered muscle recruitment that sometimes consequently induces an altered movement trajectory^2^. Compensation can be of clinical interest as an early indicator of progressive physical decline. The capacity-compensation relationship is however not fully understood. For example, compensation due to a lack of muscular capacity requires physical training, while compensation for altered sensory integration could be mitigated with skills training or technical aids. For this targeted treatment it is important to gain insight into how humans solve the functional redundancy problem as they are ageing.

A vital daily life activity for independent living is standing up, which adults conduct approximately 60 times each day^3^. The inability to stand up (e.g. from a seat or toilet) is associated with falls, frailty and institutional living^4^. Therefore this full-body motor control movement is an important movement to study in the context of capacity and compensation. Whilst sit-to-stand has been studied extensively in elderly adults, restricted experimental setups make current results inappropriate for translation to the study of capacity and compensation^5^. For example, few published studies allow the use of arms (push-off), which poorly reflects the high prevalence of this applied strategy in daily life; furthermore, foot positioning of participants is mostly fixed in a symmetrical position, shoulder width apart, with the knee angle at 90°, and foot movement during the trial is restricted. Most studies have evaluated standing-up with standing as an end-goal, rather than sit-to-walk (STW) with walking as an end-goal, while STW in daily life is more common. Lastly, age-related declining factors in capacity that have been analysed in association with the standing up movement have mostly been in isolation, excluding confounding causes within capacity and/or movement objectives. These isolated factors cannot explain why elderly adults move differently to young adults.

The aim of this study was to identify compensatory strategies in a vital daily life activity and relate these to detailed capacity measures in a group of young and elderly adults. Therefore, natural compensation strategies were analysed in the sit-to-walk task for younger (20–35 years) and (pre-frail) elderly (>65 years) adults. Aspects of the neuromuscular capacity that are known to decline with age^2^ and feasible to quantify were assessed: participants’ upper- and lower limb strength, nerve conduction, reflexes, joint sense acuity, joint range of motion, and balance. Also, standardized questionnaires on fear of falling, pain, dizziness, lifestyle, and frailty were included to identify movement objectives. Kinematics, kinetics, and muscle activity during sit-to-walk tasks were recorded in an unrestricted experimental set-up, thus permitting compensation to occur, and joint kinetics were quantified using biomechanical models.

## Results

This section first reports on the capacity differences between the age-sex groups, followed by the differences in capacity between STW strategy groups, for arm use, foot positioning, and pacing.

### Age-sex groups

Results of the peak isokinetic strength, nerve conduction study, JROM, proprioceptive acuity, balance, and FES are presented as group data for the four age-sex groups in Table 1. As expected, there are significant strength differences between YM and YW for the knee, hip, and elbow. YM also have significantly higher strength measures than EM and EW; YW have higher strength measures for the knee and hip compared to the EW, but it is notable that the strength measures of YW were not significantly different from EM. Handgrip strength was similarly different between groups: YM had significantly higher HGS than all other groups. YW had higher HGS than the EW, but, again, not than EM. EM had significantly higher HGS than the EW (YM > YW = EM > EW).

**Table 1:** Capacity measures per age-sex group.

				YW	YM	EW	EM	YW-YM	EW-EM	YW-EW	YW-EM	YM-EW	YM-EM
General data				Mean (sd)	Mean (sd)	Mean (sd)	Mean (sd)						
Age (years)				27.1 (5)	27.3 (4.3)	75 (5.6)	76.8 (7.2)			***	***	***	***
Height (cm)				168.5 (7.4)	181.5 (9.2)	161.5 (5.7)	174.4 (7.6)	***	**			***	
Bodymass (kg)				64 (8.9)	77.6 (11.6)	66 (12.5)	77.4 (13.4)	*			*		
BMI (kg/m^2^)				22.6 (3.1)	23.5 (2.7)	25.2 (3.8)	25.5 (4.3)						
TUG (s)				5.4 (0.8)	5.5 (1)	7.7 (1.5)	7.9 (2.7)			**	**	**	**
FES (-)				7 (0)	8.1 (2.5)	8.8 (2.4)	9.5 (4.3)						
Edmonton score (-)				–	–	–	–						
Healthy (-)				2.4 (0.5)	2.5 (1.2)	2.4 (1.4)	2 (0.4)						
TSTEP (s)	SELF			1.49 (0.24)	1.52 (0.22)	1.48 (0.17)	1.53 (0.33)						
	FAST			1.02 (0.14)	0.96 (0.2)	1.06 (0.11)	1.08 (0.18)						
**Strength measures (N/BW)**													
Knee	60°/s	ext.	D	1.41 (0.51)	2.48 (0.7)	0.86 (0.33)	1.13 (0.37)	***		*		***	***
			ND	1.46 (0.49)	2.47 (0.42)	0.9 (0.4)	1.26 (0.41)	***		**		***	***
		flex.	D	0.91 (0.28)	1.42 (0.32)	0.51 (0.17)	0.76 (0.24)	***		**		***	***
			ND	0.98 (0.19)	1.45 (0.26)	0.53 (0.19)	0.81 (0.28)	***	*	***		***	***
	90°/s	ext.	D	1.12 (0.52)	1.96 (0.61)	0.7 (0.35)	0.8 (0.31)	***				***	***
			ND	1.13 (0.56)	2.04 (0.44)	0.65 (0.35)	0.92 (0.37)	***		*		***	***
		flex.	D	0.69 (0.25)	1.23 (0.31)	0.38 (0.14)	0.53 (0.28)	***		*		***	***
			ND	0.75 (0.27)	1.21 (0.3)	0.42 (0.17)	0.65 (0.25)	***		*		***	***
Hip	60°/s	ext.	D	1.48 (0.54)	2.19 (0.57)	0.72 (0.31)	1.52 (0.62)	**	**	**		***	*
			ND	1.5 (0.67)	2.34 (0.56)	0.79 (0.37)	1.42 (0.63)	**		*		***	**
		flex.	D	0.83 (0.19)	1.27 (0.29)	0.37 (0.15)	0.6 (0.23)	***		***		***	***
			ND	0.79 (0.22)	1.25 (0.24)	0.39 (0.16)	0.59 (0.26)	***		***		***	***
	90°/s	ext.	D	1.11 (0.56)	1.71 (0.4)	0.42 (0.24)	0.96 (0.33)	**	*	***		***	***
			ND	1.06 (0.51)	1.71 (0.66)	0.5 (0.31)	1.08 (0.5)	*		*		***	*
		flex.	D	0.67 (0.24)	1.08 (0.27)	0.27 (0.13)	0.49 (0.18)	***		***		***	***
			ND	0.66 (0.27)	0.98 (0.36)	0.28 (0.08)	0.46 (0.25)	*		**		***	***
Ankle	60°/s	ext.	D	0.74 (0.36)	0.83 (0.32)	0.45 (0.19)	0.56 (0.29)					*	
			ND	0.69 (0.36)	0.85 (0.33)	0.51 (0.19)	0.57 (0.27)					*	
		flex.	D	0.25 (0.08)	0.33 (0.1)	0.15 (0.06)	0.19 (0.06)	*		*		***	***
			ND	0.21 (0.06)	0.26 (0.06)	0.15 (0.05)	0.19 (0.07)					***	
	90°/s	ext.	D	0.42 (0.24)	0.51 (0.22)	0.29 (0.12)	0.35 (0.15)					*	
			ND	0.44 (0.26)	0.58 (0.23)	0.32 (0.15)	0.38 (0.2)					*	
		flex.	D	0.19 (0.04)	0.27 (0.08)	0.15 (0.06)	0.18 (0.07)	**				***	**
			ND	0.17 (0.04)	0.23 (0.05)	0.16 (0.08)	0.16 (0.05)					*	
Elbow	60°/s	ext.	D	0.42 (0.16)	0.63 (0.17)	0.37 (0.1)	0.55 (0.26)	*				**	
			ND	0.45 (0.15)	0.7 (0.19)	0.37 (0.1)	0.48 (0.13)	***				***	**
		flex.	D	0.34 (0.15)	0.59 (0.13)	0.27 (0.06)	0.35 (0.11)	***				***	***
			ND	0.29 (0.12)	0.54 (0.13)	0.23 (0.06)	0.34 (0.09)	***				***	***
	90°/s	ext.	D	0.35 (0.12)	0.53 (0.13)	0.27 (0.11)	0.43 (0.17)	**	*			***	
			ND	0.39 (0.11)	0.58 (0.16)	0.28 (0.08)	0.36 (0.11)	***				***	***
		flex.	D	0.31 (0.12)	0.57 (0.12)	0.2 (0.06)	0.33 (0.12)	***	*	*		***	***
			ND	0.25 (0.1)	0.5 (0.13)	0.23 (0.07)	0.3 (0.11)	***				***	***
**Handgrip strength (Kg)**													
HGS			D	28.71 (8.89)	49 (9.09)	20.21 (4.76)	33.55 (7.54)	***	**	*		***	***
			ND	27.43 (6.79)	46.31 (10.3)	18.04 (4.28)	33.82 (7.6)	***	***	*		***	**
**Balance score (Z-score)**													
AP			EO	0.26 (0.37)	−0.28 (0.55)	−0.12 (1.1)	0.13 (0.97)						
			EC	0.08 (0.39)	−0.21 (0.51)	−0.11 (1.05)	0.26 (0.78)						
ML			EO	0.07 (0.69)	−0.13 (0.61)	−0.09 (1.3)	0.15 (0.43)						
			EC	−0.01 (0.37)	−0.25 (0.32)	−0.05 (1.06)	0.3 (0.87)						
**Proprioception (deg)**													
Maximum error			D	6.68 (2.21)	7.22 (4.47)	7.53 (3.07)	7.79 (2.98)						
			ND	7.66 (2.92)	7.1 (2.28)	7.21 (4.82)	7.09 (2.46)						
Minimum error			D	0.71 (0.82)	0.59 (0.53)	0.65 (0.38)	1.09 (1.39)						
			ND	0.73 (0.65)	0.36 (0.38)	0.82 (1.47)	0.75 (0.99)						
Mean error			D	2.98 (1.05)	2.89 (1.59)	3.35 (1.05)	3.87 (2.24)						
			ND	3.49 (1.66)	2.82 (0.97)	3.36 (2.8)	3.37 (1.39)						
s.d. error			D	1.87 (0.55)	2.16 (1.4)	2.31 (0.94)	2.19 (0.65)						
			ND	2.33 (0.98)	2.14 (0.58)	2.02 (1.17)	2.13 (0.66)						
**Joint Range of Motion (deg)**													
Hip	flex.		D	135.43 (9.17)	136.31 (5.88)	128.42 (4.08)	123.8 (11)				**		**
			ND	134.79 (9.5)	134.15 (4.98)	127.83 (5.15)	126.6 (9.08)						
Ankle	pflex.		D	64.29 (12.08)	54.31 (8.57)	56.17 (11.54)	39.4 (11.75)		**		***		*
			ND	64.86 (7.44)	57.62 (10.38)	56.17 (10.88)	40.8 (14.94)		**		***		**
	dflex.		D	−36.57 (7.14)	−35.77 (9.82)	−32.75 (8.52)	−28.4 (11.12)						
			ND	−36.21 (11.14)	−34.92 (9.2)	−31.5 (6.88)	−28.6 (6.82)						
**Nerve conduction study**													
CMAP (mV)	median			6.47 (5.02)	10.41 (4.03)	5.25 (2.42)	5.82 (3.53)					*	
	tibial			11.78 (2.69)	15.57 (4.01)	8.87 (4.26)	8.97 (4.24)					**	**
	peroneal			4.7 (2.65)	6.45 (2.25)	2.95 (1.21)	3.23 (1.71)					**	**
PMCT (ms/cm)	median			0.09 (0.01)	0.09 (0)	0.1 (0.01)	0.1 (0.01)			*		*	
	tibial			0.1 (0.01)	0.1 (0.01)	0.11 (0)	0.12 (0)			*	***	*	**
	peroneal			0.16 (0.01)	0.15 (0)	0.17 (0.02)	0.17 (0.01)					*	**
Hlat. (ms/cm)	tibial			0.18 (0.01)	0.17 (0.01)	0.2 (0.02)	0.2 (0.02)					**	**

*ext* extension, *flex* flexion, *D* dominant side, *ND* non-dominant side, *EO* eyes open, *EC* eyes closed.

*p* value (ANOVA): *=*p* < 0.05, **=*p* < 0.01, ***=*p* < 0.001.

CMAP amplitude and latency values captured are comparable to normative data^6,7^. H-reflex latency for all participants was 31.7 ± 3.1 ms (young: 30.2 ± 2.5 ms, old: 33.3 ± 3 ms), which is in line with normative data^8^. The mean CMAP of YM were higher than those of EW for all tested sites, and higher than EM for the tibial and peroneal nerve (Table 1). One elderly man had a much higher CMAP amplitude (14.3 mV) for the median nerve compared to the rest of EM (range: 2.4–7 mV); when this participant was not included, the mean CMAP at the median nerve was higher for YM than EM. There was no significant difference between the means of YW and YM, nor between EW and EM.

Ankle plantarflexion JROM was significantly smaller for EM compared to the other three groups both on the dominant and the non-dominant side (Table 1). Mean hip flexion ROM for EM was smaller than YW and YM on the dominant side (Table 1).

Results of the proprioceptive acuity test did not show significant differences in knee joint position sense acuity between the four groups. There were three elderly participants that had a particularly higher error in the proprioceptive test compared to the other elderly participants, which could be explained by their respective history of a knee replacement and diabetes, osteoarthritis, and knee infections.

From the balance test, no differences were found between the four groups for the composite z-score for the eyes open and closed condition for the AP and ML directions (Table 1). There were six participants (3EM, 2EW, 1YM) with an FES score of >10, indicating a high fear of falling. Based on their medical history, two of these participants had a history of falls (1EM, 1EW), two indicated recent episodes of dizziness (1EM,1EW), and one recovered from a stroke (1EM). One of the subjects with a high FES score was a younger man; he indicated a fear of slipping in the bathroom or falling down the stairs; based on his medical history we could not find a direct cause for this fear, however, this participant did report reoccurring episodes of stress and anxiety in the medical history section.

### Sit-to-walk strategies

#### Arm strategies

##### Compensation strategies

**Fig. 1:**
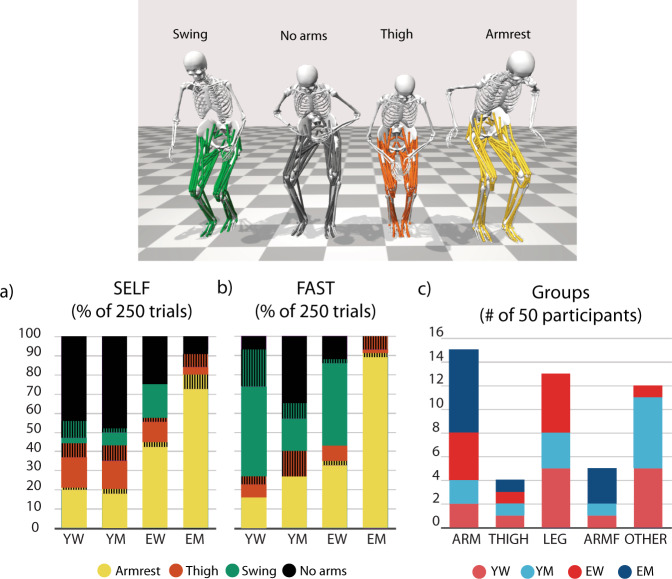
Arm related compensation strategies in the sit-to-walk trials at self-selected speed (SELF) and fast speed (FAST). **a**, **b** Percentage of trials in which participants, grouped by age-sex, used a particular arm strategy. Patterned blocks indicate that each arm was doing something different (asymmetric strategy). **c** Number of participants per arm strategy group. Participants were divided into five arm strategy groups based on the consistency of arm use.

Arm strategies were analysed for each participant, per age-sex group, and for all trials (250 trials per condition) (Fig. 1). Note that all participants were able to conduct the task without use of armrests, or thigh push-off. Of note is the number of trials in which EM used an arm push-off to stand up, either on the armrest or on the thighs: 93% in SELF, and 100% in FAST (Fig. 1b). Overall, the thigh push-off was used in 11% of all trails in SELF and 4% in FAST by 13 different participants.

Based on the arm strategies, participants were divided into five arm strategy groups (Fig. 1c):

- *armrest only (ARM):* 15 participants (2 YW, 2 YM, 4 EW, 7 EM) who consistently pushed-off on the armrests in all trials, both conditions.
- *Thigh push off only (THIGH)*: 4 participants (1YW, 1YM, 1EW, 1EM) who consistently pushed of on their thighs in all trials, both conditions.
- *no arms (LEG):* 13 participants (5YW, 3YM, 5EW) who never used their arms to push-off in any of the trials, both conditions; these participants either swung their arms or did not use their arms at all.
- *arms in F (ARMF)*: 5 participants (1YW, 1YM, 3EM) who consistently used their arms to push-off in all trails in FAST, but were inconsistent in SELF.
- *others (OTHER)*: 13 participants (5YW, 6YM, 1EW) who were inconsistent in the use of arm push-off in both conditions.

The characteristics of these arm strategy groups are shown in Table 2. The ARM group was on average significantly older than the OTHER group and slower in TUG: 63.5 versus 35.1 years, and 7.6 versus 5.6 seconds respectively. There were no other significant differences in age, weight, height, BMI, TUG time, frailty or health score between these groups (Table 2).

**Table 2:** Capacity measures per arm strategy group.

				ARM (*N* = 15)	THIGH (*N* = 4)	ARMF (*N* = 5)	LEG (*N* = 13)	OTHER (*N* = 13)
General data				Mean (sd)	Mean (sd)	Mean (sd)	Mean (sd)	Mean (sd)
Age (years)				**63.5 (25.9)a**	50.5 (30.1)	52.8 (22.8)	46.5 (24.1)	**35.1 (18)a**
Height (cm)				172.7 (11.7)	167 (7.3)	181.3 (5.3)	168.5 (10)	170.8 (10.3)
Bodymass (kg)				74.9 (12.7)	68 (13.4)	81.1 (10.6)	67.8 (14.8)	66.6 (9.8)
BMI (kg/m^2^)				25.3 (4.7)	24.3 (3.9)	24.7 (3.4)	23.7 (3.1)	22.8 (2.4)
TUG (s)				**7.6 (2.9)a**	7.4 (1.4)	5.7 (0.7)	6.3 (1.2)	**5.6 (0.9)a**
FES (-)				9.5 (4.1)	**12.7 (3.5)b,c,d**	**7.2 (0.4)b**	**7.3 (0.7)c**	**7.4 (0.8)d**
Edmonton Score (-)				2.2 (2.2)	2 (1.4)	0.7 (1.2)	1.8 (0.8)	0 (0)
Healthy (-)				2.4 (1.2)	2.8 (1)	2.4 (0.5)	2.2 (0.9)	2.2 (0.9)
TSTEP (s)	SELF			1.5 (0.3)	1.6 (0.2)	1.4 (0.1)	1.5 (0.2)	1.5 (0.2)
	FAST			1.1 (0.2)	1.1 (0.1)	1 (0.1)	1 (0.1)	1 (0.2)
**Strength measures (N/BW)**								
Knee	60°/s	ext.	D	1.27 (0.74)	1.49 (0.64)	1.74 (0.87)	1.45 (0.64)	1.73 (1.02)
			ND	1.29 (0.69)	1.31 (0.76)	1.64 (0.65)	1.64 (0.58)	1.78 (0.91)
		flex.	D	0.79 (0.46)	0.86 (0.24)	1.05 (0.39)	0.83 (0.33)	1.1 (0.5)
			ND	0.81 (0.46)	0.84 (0.33)	1.12 (0.34)	0.97 (0.3)	1.09 (0.47)
	90°/s	ext.	D	1.02 (0.67)	1.07 (0.54)	1.24 (0.88)	1.2 (0.57)	1.33 (0.82)
			ND	1.05 (0.71)	1.08 (0.61)	1.23 (0.61)	1.31 (0.6)	1.33 (0.84)
		flex.	D	0.64 (0.45)	0.59 (0.24)	0.79 (0.5)	0.67 (0.29)	0.89 (0.46)
			ND	0.67 (0.43)	0.7 (0.28)	0.94 (0.33)	0.77 (0.28)	0.84 (0.47)
Hip	60°/s	ext.	D	1.4 (0.9)	1.31 (0.28)	2.22 (0.4)	1.39 (0.6)	1.46 (0.77)
			ND	1.35 (0.86)	1.31 (0.43)	2.26 (0.47)	1.42 (0.63)	1.65 (0.93)
		flex.	D	0.67 (0.52)	0.71 (0.21)	0.91 (0.17)	0.76 (0.33)	0.91 (0.42)
			ND	0.68 (0.51)	0.73 (0.25)	0.97 (0.23)	0.74 (0.3)	0.84 (0.42)
	90°/s	ext.	D	0.94 (0.72)	0.94 (0.29)	1.49 (0.4)	1 (0.56)	1.16 (0.67)
			ND	1.03 (0.81)	0.88 (0.12)	1.74 (0.71)	1 (0.43)	1.11 (0.71)
		flex.	D	0.56 (0.45)	0.56 (0.26)	0.78 (0.31)	0.62 (0.3)	0.73 (0.39)
			ND	0.59 (0.51)	0.52 (0.17)	0.78 (0.29)	0.56 (0.23)	0.65 (0.37)
Ankle	60°/s	ext.	D	0.57 (0.43)	0.44 (0.13)	0.88 (0.32)	0.67 (0.14)	0.71 (0.34)
			ND	0.63 (0.44)	0.46 (0.15)	0.87 (0.32)	0.63 (0.17)	0.72 (0.3)
		flex.	D	0.18 (0.08)	0.22 (0.06)	0.22 (0.06)	0.24 (0.09)	0.29 (0.13)
			ND	0.18 (0.06)	0.21 (0.1)	0.22 (0.08)	0.18 (0.05)	0.24 (0.07)
	90°/s	ext.	D	0.38 (0.28)	0.33 (0.14)	0.49 (0.2)	0.38 (0.1)	0.42 (0.23)
			ND	0.39 (0.32)	0.37 (0.2)	0.55 (0.28)	0.41 (0.13)	0.49 (0.19)
		flex.	D	0.19 (0.08)	0.18 (0.05)	0.19 (0.04)	0.2 (0.07)	0.23 (0.09)
			ND	0.17 (0.08)	0.19 (0.08)	0.2 (0.05)	0.16 (0.04)	0.21 (0.07)
Elbow	60°/s	ext.	D	0.5 (0.21)	0.4 (0.1)	0.62 (0.2)	0.46 (0.13)	0.49 (0.26)
			ND	0.47 (0.18)	0.46 (0.11)	0.6 (0.09)	0.5 (0.16)	0.52 (0.27)
		flex.	D	0.32 (0.16)	0.46 (0.17)	0.51 (0.13)	0.37 (0.12)	0.41 (0.22)
			ND	0.31 (0.14)	0.42 (0.12)	0.47 (0.12)	0.32 (0.15)	0.35 (0.2)
	90°/s	ext.	D	0.39 (0.16)	0.26 (0.09)	0.5 (0.15)	0.38 (0.11)	0.41 (0.2)
			ND	0.35 (0.16)	0.39 (0.11)	0.47 (0.11)	0.41 (0.13)	0.45 (0.22)
		flex.	D	0.29 (0.17)	0.4 (0.14)	0.5 (0.14)	0.34 (0.14)	0.36 (0.22)
			ND	0.28 (0.13)	0.39 (0.1)	0.42 (0.15)	0.31 (0.13)	0.32 (0.19)
**Handgrip strength (Kg)**								
HGS			D	32.4 (11.69)	36 (18.96)	41.2 (7.43)	30.69 (13.18)	31.96 (14.81)
			ND	31.67 (11.42)	34.5 (16.03)	38 (9.38)	27.27 (14.08)	32.08 (13.39)
**Balance score (Z-score)**								
Anterior-Posterior			EO	0.16 (0.91)	0.38 (0.27)	−0.1 (0.47)	−0.24 (1)	−0.05 (0.6)
			EC	0.23 (0.68)	−0.01 (0.72)	−0.24 (0.46)	−0.22 (1.02)	0.03 (0.37)
Medial-Lateral			EO	0.05 (0.49)	0.03 (0.34)	−0.06 (0.37)	−0.14 (1.36)	0.08 (0.63)
			EC	0.13 (0.81)	0.09 (0.63)	−0.15 (0.41)	−0.18 (1)	−0.01 (0.24)
**Proprioception (deg)**								
Mean error			D	**2.71 (0.8)e**	3.17 (1.38)	**5.17 (2.72)e,f**	**2.98 (0.98)f**	3.38 (1.69)
			ND	2.63 (0.83)	4.41 (2.17)	4.31 (1.49)	3.75 (2.72)	2.76 (0.9)
**Joint Range of Motion (deg)**								
Hip	flex.		D	**125.71 (9.71)a**	133.25 (6.65)	133.6 (8.08)	**133.69 (6.17)a**	134.46 (10.2)
			ND	128 (9.06)	133.25 (4.11)	134.4 (7.06)	130.15 (6.36)	134 (9.21)
Ankle	pflex.		D	**46.5 (12.28)a**	58.75 (10.69)	55.6 (24.63)	**58.62 (10.81)a**	57.54 (11.86)
			ND	**47.79 (10.82)A**	54.25 (13.6)	49 (24.58)	**63.92 (8.99)A**	59.77 (9.58)
	dflex.		D	−29.14 (10.09)	−29.75 (4.65)	−37.2 (8.87)	−33.54 (8.51)	−38.85 (8.48)
			ND	−28.64 (7.4)	−32.5 (5.74)	−36.4 (9.37)	−32.23 (10.98)	−37.92 (7.77)
**Nerve conduction study**								
CMAP (mV)	median			8.37 (4.8)	4.56 (2.04)	5.96 (1.53)	5.27 (3.06)	7.81 (4.85)
	tibial			10.18 (4.12)	13.56 (4.19)	7.52 (3.04)	10.35 (3.72)	14.32 (5.27)
	peroneal			4.26 (2.32)	7.06 (4.08)	3.72 (1.99)	4.33 (2.81)	4.12 (1.76)
PMCT (ms/cm)	median			0.1 (0.01)	0.08 (0)	0.1 (0)	0.1 (0.01)	0.09 (0.01)
	tibial			0.11 (0.01)	0.11 (0.01)	0.11 (0)	0.11 (0.01)	0.11 (0.01)
	peroneal			0.16 (0.01)	0.16 (0.02)	0.17 (0.01)	0.16 (0.02)	0.15 (0.01)
Hlat. (ms/cm)	tibial			0.18 (0.02)	0.19 (0.03)	0.19 (0.01)	0.18 (0.02)	0.18 (0.02)

In bold significant differences in means between (ANOVA): A = ARM and OTHER (*p* < 0.001); a = ARM and OTHER (*p* < 0.05); b = THIGH and ARMF (*p* < 0.05); c = THIGH and LEG (*p* < 0.05); d = THIGH and OTHER (*p* < 0.05); e = ARM and ARMF (*p* < 0.05); f = LEG and ARMF (*p* < 0.05).

##### Capacity

No differences were found between the means of the arm-groups for any of the peak isokinetic joint moment measures, handgrip strength, CMAP amplitude, or H-reflex and PMCT latencies (Table 2). The ARMF group had a significantly higher average mean knee joint sense acuity error on the dominant side compared the other groups; however, this is explained by a significantly higher score of one elderly man in ARMF who had a knee replacement on the dominant side, and suffers from diabetes.

**Fig. 2:**
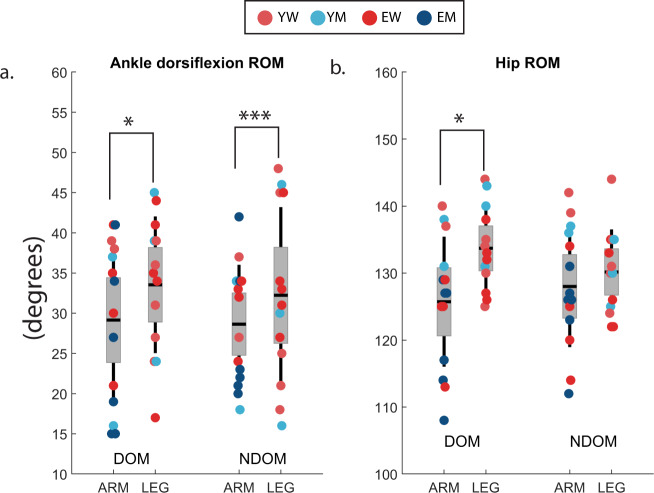
Joint range of motion capacity versus arm strategies. Joint range of motion of (**a**) Ankle dorsiflexion and (**b**) hip flexion within the ARM and LEG groups for the dominant (DOM) and non-dominant (NDOM) sides. LEG has larger ROM than ARM; *p < 0.05, ***p < 0.001 (*t* test); shown are the 95% confidence interval for the mean (1.96 s.e.m), lines indicate standard deviation (s.d.).

There were significant differences in the JROM. ARM had a lower ankle plantar-flexion range of motion on the non-dominant side compared to LEG (*p* < 0.01, mean = 48 deg vs 64 deg). ARM and LEG can be considered the most homogeneous and distinct groups, therefore statistics were rerun comparing just the ARM group to the LEG group (excluding the THIGH, ARMF and OTHER) (Fig. 2). This showed a significant lower ankle plantar-flexion range of motion on the non-dominant side (*p* < 0.001, mean = 48 deg vs 64 deg), and the dominant-side (*p* < 0.05, 47 deg vs 59 deg) for ARM. Additionally, a significant lower hip flexion range of motion was found on the dominant side for ARM (*p* < 0.05, mean = 126 deg vs 134 deg). The hip flexion range of motion of ARM on the non-dominant side was also lower, but not significantly. The results for all other capacity measures remained the same. There was no difference between the means of the groups for the composite balance scores. Interestingly however, all participants with a FES score >10, meaning they were very afraid of falling, were in either the ARM (2EM, 2EW) or the THIGH (1EM, 1YM) group.

##### Task demand

**Fig. 3:**
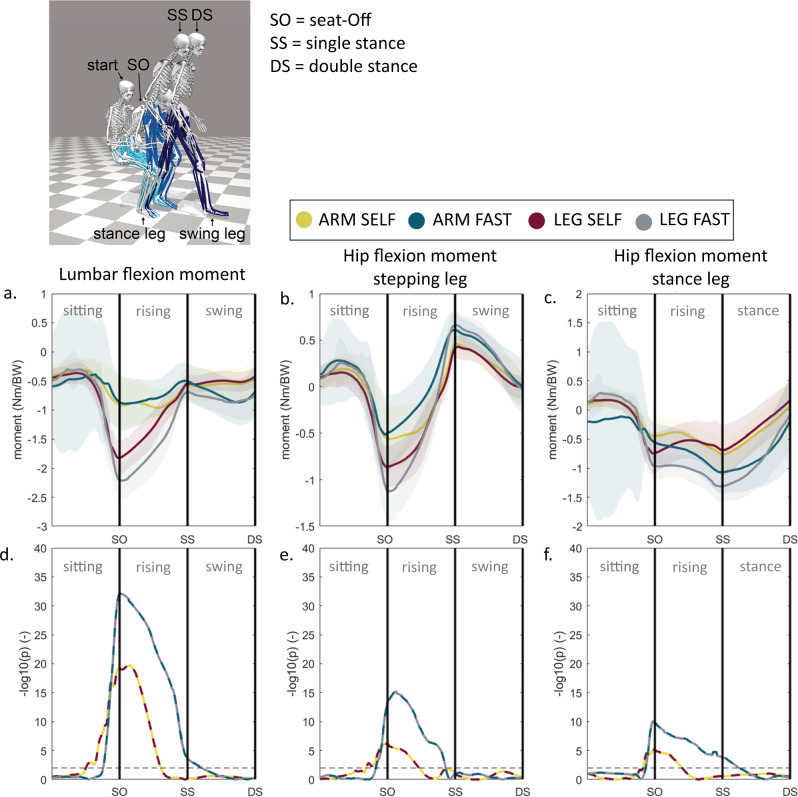
Joint moment during sit-to-walk for distinct arm strategies. Trajectory comparison between ARM and LEG of the (**a**) lumbar and (**b**, **c**) hip joint moments corrected by body weight (BW). The stepping leg is the leg that steps out first. **d**–**f** Trajectory analysis: *t* tests were performed for every time sample. The level of significance is visualized as the negative base-10 logarithm of the *p* value so that large values represent small p-values; black dashed horizontal line indicates a significant difference (*p* < 0.01).

**Fig. 4:**
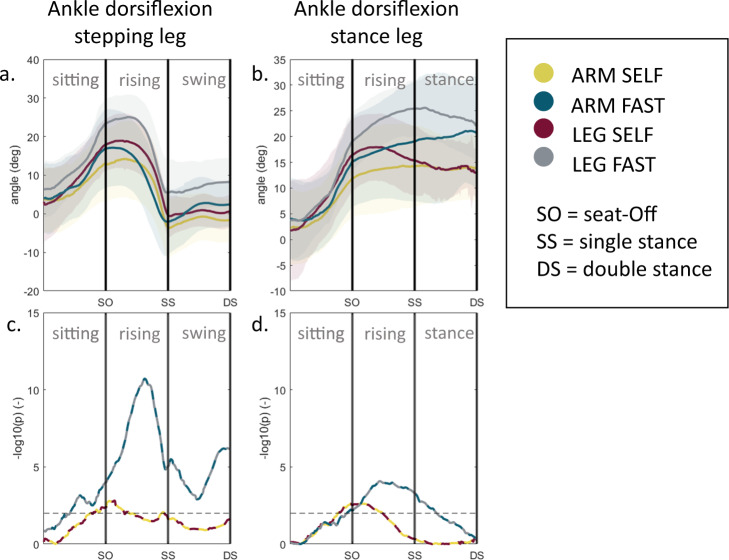
Joint angle trajectories during sit-to-walk for distinct arm strategies. **a**, **b** Trajectory comparison between ARM and LEG of the ankle joint angles. The stepping leg is the leg that steps out first. **c**, **d** Trajectory analysis: *t* tests were performed for every time sample. The level of significance is visualized as the negative base-10 logarithm of the *p* value so that large values represent small *p* values; black dashed horizontal line indicates a significant difference (*p* < 0.01).

ARM significantly differs from the other strategy groups in joint moments and angles. ARM and LEG are most distinct, therefore the trajectory analysis of these groups are visualised in Figs. 3, 4. The lumbar extension moment (Fig. 3a) is significantly lower for ARM compared to LEG prior to seat-off until halfway of the standing up phase in SELF, and throughout the standing up phase in FAST. The hip moments (Fig. 3b, c) show a similar difference trend. In SELF, ARM on average had less dorsi-flexion in the ankle than LEG prior to seat-off and just after seat-off (Fig. 4). In FAST there is significantly less dorsiflexion throughout the standing up phase.

#### Foot placement

##### Compensation strategies

The foot strategies are expressed by the normalised width of the base-of-support (cML-BOS) and the normalised distance (asymmetry) between the feet in the anterior-posterior position (cAP-BOS). There was no difference of means of cML-BOS or cAP-BOS between SELF and FAST. Comparing the age-sex groups, YM had a significantly wider cML-BOS than the other three groups (Table 3). There was no difference in cML-BOS or cAP-BOS between the arm strategy groups.

**Table 3:** Capacity measures versus the foot positioning (*N* = 50).

				cML-BOS		cAP-BOS	
General data				SELF	FAST	SELF	FAST
Age (yrs)				*R* = *0.12, b* = −*0.01*	*R* = *0.13, b* = −*0.01*	R = 0.08, b = 0	
Height (cm)				*R* = *0.15, b* = *0.01*	**R** = **0.21, b** = **0.01**		
Bodymass (kg)							
BMI (*kg*/*m*^2^)							
TUG (s)							
FES (-)							
Edmonton Frailty Score (-)							
Healthy (-)							
TSTEP (s)		SELF					
		FAST					
**Strength measures (N/BW)**							
Knee	60°/s	ext.	D	**R** = **0.31, b** = **0.26**	**R** = **0.28, b** = **0.22**		
			ND	**R** = **0.35, b** = **0.3**	**R** = **0.29, b** = **0.25**		
		flex.	D	**R** = **0.46, b** = **0.58**	**R** = **0.5, b** = **0.54**		
			ND	**R** = **0.46, b** = **0.61**	**R** = **0.45, b** = **0.53**		
	90°/s	ext.	D	**R** = **0.34, b** = **0.32**	**R** = **0.35, b** = **0.29**		
			ND	**R** = **0.41, b** = **0.34**	**R** = **0.36, b** = **0.3**		
		flex.	D	**R** = **0.47, b** = **0.61**	**R** = **0.5, b** = **0.57**		
			ND	**R** = **0.52, b** = **0.68**	**R** = **0.49, b** = **0.61**		
Hip	60°/s	ext.	D	**R** = **0.3, b** = **0.28**	**R** = **0.34, b** = **0.27**		
			ND	**R** = **0.25, b** = **0.24**	**R** = **0.31, b** = **0.23**		
		flex.	D	**R** = **0.48, b** = **0.64**	**R** = **0.46, b** = **0.57**		
			ND	**R** = **0.36, b** = **0.57**	**R** = **0.37, b** = **0.54**		
	90°/s	ext.	D	**R** = **0.26, b** = **0.31**	**R** = **0.28, b** = **0.31**		
			ND	**R** = **0.26, b** = **0.29**	**R** = **0.29, b** = **0.27**		
		flex.	D	**R** = **0.46, b** = **0.67**	**R** = **0.42, b** = **0.59**		
			ND	**R** = **0.37, b** = **0.61**	**R** = **0.4, b** = **0.59**		
Ankle	60°/s	ext.	D	R = 0.08, b = 0.34	R = 0.08, b = 0.35		
			ND				
		flex.	D	**R** = **0.22, b** = **1.71**	*R* = *0.17, b* = *1.38*		
			ND				
	90°/s	ext.	D	R = 0.12, b = 0.64	R = 0.11, b = 0.65		
			ND				
		flex.	D	**R** = **0.27, b** = **2.55**	*R* = *0.18, b* = *1.9*		
			ND				
Elbow	60°/s	ext.	D	**R** = **0.22, b** = **0.88**	**R** = **0.3, b** = **0.9**		R = 0.08, b = −0.12
			ND	**R** = **0.37, b** = **1.18**	**R** = **0.36, b** = **1.04**		
		flex.	D	**R** = **0.4, b** = **1.34**	**R** = **0.34, b** = **1.13**		
			ND	**R** = **0.28, b** = **1.2**4	**R** = **0.24, b** = **1.04**		
	90°/s	ext.	D	**R** = **0.28, b** = **1.25**	**R** = **0.33, b** = **1.2**		
			ND	**R** = **0.44, b** = **1.49**	**R** = **0.41, b** = **1.3**		
		flex.	D	**R** = **0.37, b** = **1.29**	**R** = **0.33, b** = **1.1**1		
			ND	**R** = **0.29, b** = **1.31**	**R** = **0.26, b** = **1.15**	R = 0.07, b = −0.06	
**Handgrip Strength (kg)**							
Handgrip Strength			D	**R** = **0.3, b** = **0.02**	**R** = **0.31, b** = **0.01**		
			ND	**R** = **0.27, b** = **0.02**	**R** = **0.3, b** = **0.01**		
**Balance score (Z-score)**							
Anterior-Posterior			EO				
			EC				
Medial-Lateral			EO				R = 0.09, b = −0.03
			EC				
**Proprioception (deg)**							
mean error			D				
			ND				
**Joint Range of Motion**							
Hip	flex.		D				
			ND				
Ankle	plantarflex.		D				*R* = *0.13, b* = *0*
			ND			R = 0.07, b = 0	
	dorsiflex.		D				
			ND				
**Nerve Conduction Study**							
CMAP (mV)	median			R = 0.13, b = 0.04	R = 0.09, b = 0.03		
	tibial			*R* = *0.19, b* = *0.04*	*R* = *0.18, b* = *0.03*		
	peroneal			**R** = **0.35, b** = **0.1**	**R** = **0.36, b** = **0.09**		
PMCT (ms/cm)	median						
	tibial			R = 0.17, b = −20.15			
	peroneal			*R* = *0.21, b* = −*15.1*9	*R* = *0.2, b* = −*13.12*		
Hlat (ms)	tibial			*R* = *0.22, b* = −*9.45*	*R* = *0.24, b* = −*9.28*		

The adjusted *R*^2^ (*R*) and normalized slope of the linear regression (*b*) are provided if a significant regression was found (*p* < 0.05). *Italic* indicates regressions with *p* < 0.01, in bold regressions with *p* < 0.001.

##### Capacity

**Fig. 5:**
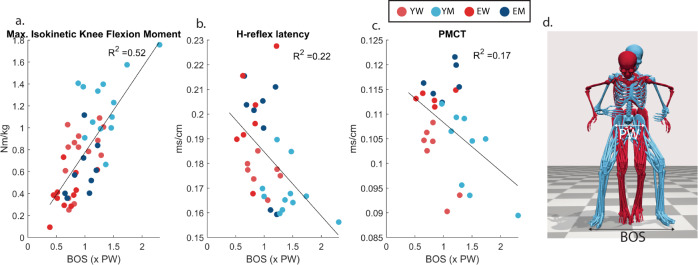
Base-of-support (foot positioning) versus strength and nerve conduction measures. Linear regressions of BOS versus **a** maximal isokinetic knee flexion strength (*p* < 0.001). **b** H-reflex latency (*p* = 0.003). **c** PMCT-tibial nerve (*p* = 0.02). **d** 3D motion capture visualization of a small BOS versus a wide BOS of a YM (blue) versus an EW (red) during the rising phase.

There were several significant linear relationships (regressions) between the isokinetic strength measures and the (c)ML-BOS foot positioning (Table 3). Participants with higher knee and hip strength measures used a wider foot positioning in standing up, with a larger correlation to flexion than extension (Fig. 5a). The same positive relationship was also found for the elbow strength, but without the difference of effects between flexion and extension. For the ankle strength the positive relationship between cML-BOS was only significant on the dominant side and smaller compared to the knee and hip (Table 3). HGS also showed a positive linear regression with the cML-BOS on both sides (Table 3).

In line with these strength measures, the results showed a small significant positive linear regression between CMAP and cML-BOS for the peroneal, tibial, and median nerve, indicating that participants with a higher maximum CMAP had a wider cML-BOS (Table 3). There was also a significant negative relationship between cHlat and cML-BOS, indicating that participants with a longer latency used a smaller cML-BOS (Fig. 5b). This was also found for the cPMCT-latency, with a small statistically significant relationship, for the tibial and peroneal nerve (Table 3) (Fig. 5c). There was no significant linear regression between cAP-BOS and CMAP, cPMCT, or cHlat.

Length was the only other measure with a significant linear regression with cML-BOS, taller participants used a wider cML-BOS (corrected for hip width). We performed several step-wise multivariate regressions with length and one of the isokinetic strength measures as input. Length was not a strong, and for most regressions even an insignificant, predictor, indicating that strength is the stronger predictor than length.

##### Task demand

To gain insight into the differences in joint moments between participants based on the cML-BOS, results were divided into three foot-categories: small (cML-BOS < 0.8), medium (cML-BOS = 0.8–1.2), and wide (cML-BOS > 1.2) Characteristics of the groups can be found in the supplementary material (Supplementary Table 3). Around seat-off, the joint moment trajectories of the wide group differed from small and medium, small and medium did not show a clear difference; in the rising phase, participants with a wide BOS had larger hip and knee extension moments in the stepping leg in both the SELF and FAST compared to the small and medium foot-groups. There were no differences in the joint moment trajectories of the ankle joint moment. Note that the arm strategies and AP BOS positions within the foot strategy groups differ.

#### Pacing

##### Compensation strategies

To analyse pacing, TSTEP, *rTUL*, and the vertical and horizontal COM velocity and acceleration trajectories were considered. Four (age: 79, 82, 80, 92) out of 50 participants unloaded their swing foot after the peak COM vertical velocity, all other participants unloaded the swing feet prior to the peak COM velocity. Delayed unloading of the foot had small but significant relationships to higher age, longer TUG times, and higher FES scores (Table 4). In TSTEP there was one outlier, an elderly man aged 92, who moved significantly (>2x standard deviation) slower than any of the other participants (SELF: mean TSTEP = 2.28 s; FAST: mean TSTEP = 1.50 s). Therefore, for the purpose of the pacing analysis, this man was excluded. There was no significant difference in TSTEP or rTUL between age-sex groups or between arm strategy groups. Nor was there a relationship between BOS and TSTEP or rTUL. However, there were differences in the COM velocities; EW had lower VERVEL and VERACC in the rising phase compared to the other groups. To increase the movement speed in FAST, participants increased HORVEL only, not VERVEL.

**Table 4:** Capacity measures versus pacing measures (*N* = 49).

				TSTEP		HORVEL		VERVEL		rTUL	
General data				SELF	FAST	SELF	FAST	SELF	FAST	SELF	FAST
Age (years)							**R** = **0.43, b** = −**0.01**		R = 0.12, b = 0	R = 0.11, b = 0	
Height (cm)							**R** = **0.39, b** = **0.01**	**R** = **0.49, b** = **0.01**	**R** = **0.34, b** = **0.01**	R = 0.08, b = 0	
Bodymass (kg)							R = 0.18, b = 0.01	R = 0.13, b = 0	R = 0.15, b = 0		
BMI (kg/m^2^)											
TUG (s)							**R** = **0.46, b** = −**0.09**	R = 0.13, b = −0.04	R = 0.12, b = −0.03	R = 0.32, b = 0.04	
FES (-)				R = 0.12, b = 0.04			*R* = *0.27, b* = −*0.11*			R = 0.2, b = 0.02	
Edmonton (-)							R = 0.28, b = −0.06				
Healthy (-)										R = 0.08, b = 0.04	
**Strength Measures (N/BW)**											
Knee	60°/s	ext.	D		R = 0.12, b = −0.07		**R** = **0.46, b** = **0.17**	R = 0.13, b = 0.08	R = 0.11, b = 0.06		
			ND		*R* = *0.19, b* = −*0.09*		**R** = **0.56, b** = **0.21**	*R* = *0.22, b* = *0.1*	R = 0.15, b = 0.07	R = 0.1, b = −0.05	
		flex.	D				**R** = **0.62, b** = **0.38**	*R* = *0.21, b* = *0.17*	R = 0.15, b = 0.14		
			ND		*R* = *0.16, b* = −*0.16*		**R** = **0.69, b** = **0.45**	*R* = *0.25, b* = *0.2*	*R* = *0.18, b* = *0.16*		
	90°/s	ext.	D		R = 0.1, b = −0.07		**R** = **0.41, b** = **0.19**	R = 0.12, b = 0.08			
			ND		R = 0.12, b = −0.07		**R** = **0.57, b** = **0.23**	*R* = *0.22, b* = *0.11*	R = 0.17, b = 0.08	R = 0.08, b = −0.05	
		flex.	D				**R** = **0.55, b** = **0.39**	R = 0.15, b = 0.16			
			ND		*R* = *0.2, b* = −*0.18*		**R** = **0.62, b** = **0.46**	*R* = *0.21, b* = *0.2*	R = 0.12, b = 0.14		
Hip	60°/s	ext.	D		R = 0.1, b = −0.07		**R** = **0.62, b** = **0.24**	**R** = **0.4, b** = **0.14**	*R* = *0.19, b* = *0.09*		
			ND				**R** = **0.51, b** = **0.19**	**R** = **0.46, b** = **0.13**	*R* = *0.23, b* = *0.09*		
		flex.	D		R = 0.09, b = −0.12		**R** = **0.64, b** = **0.4**	*R* = *0.24, b* = *0.2*	*R* = *0.2, b* = *0.16*		
			ND				**R** = **0.55, b** = **0.39**	**R** = **0.32, b** = **0.23**	*R* = *0.28, b* = *0.18*	R = 0.07, b = −0.08	
	90°/s	ext.	D		*R* = *0.2, b* = −*0.11*		**R** = **0.63, b** = **0.28**	**R** = **0.31, b** = **0.14**	R = 0.13, b = 0.09		
			ND		R = 0.13, b = −0.09		**R** = **0.5, b** = **0.25**	**R** = **0.5, b** = **0.19**	*R* = *0.18, b* = *0.11*		
		flex.	D		*R* = *0.17, b* = −*0.17*		**R** = **0.61, b** = **0.44**	**R** = **0.29, b** = **0.25**	R = 0.14, b = 0.15		
			ND		R = 0.1, b = −0.15		**R** = **0.41, b** = **0.42**	*R* = *0.25, b* = *0.24*	*R* = *0.18, b* = *0.19*		
Ankle	60°/s	ext.	D		*R* = *0.25, b* = −*0.23*		**R** = **0.4, b** = **0.41**				
			ND		*R* = *0.18, b* = −*0.22*		*R* = *0.28, b* = *0.43*	R = 0.09, b = 0.18			
		flex.	D		*R* = *0.19, b* = −*0.67*		**R** = **0.56, b** = **1.59**		R = 0.1, b = 0.51		
			ND				**R** = **0.36, b** = **1.81**	R = 0.11, b = 0.76	R = 0.16, b = 0.86		
	90°/s	ext.	D		*R* = *0.21, b* = −*0.35*		**R** = **0.37, b** = **0.65**				
			ND	R = 0.13, b = 0.35	R = 0.15, b = −0.26		**R** = **0.39, b** = **0.63**	R = 0.14, b = 0.26			
		flex.	D		R = 0.1, b = −0.63		*R* = *0.21, b* = *1.22*		R = 0.14, b = 0.69		
			ND								
Elbow	60°/s	ext.	D				**R** = **0.37, b** = **0.53**	R = 0.13, b = 0.28	R = 0.11, b = 0.23		
			ND				**R** = **0.45, b** = **0.66**	*R* = *0.18, b* = *0.36*	R = 0.16, b = 0.29		
		flex.	D				**R** = **0.5, b** = **0.82**	*R* = *0.18, b* = *0.42*	**R** = **0.29, b** = **0.44**		
			ND				**R** = **0.42, b** = **0.83**	*R* = *0.26, b* = *0.51*	**R** = **0.32, b** = **0.5**		
	90°/s	ext.	D		R = 0.12, b = −0.3		**R** = **0.48, b** = **0.78**	*R* = *0.24, b* = *0.46*	R = 0.17, b = 0.35		
			ND		R = 0.12, b = −0.3		**R** = **0.42, b** = **0.75**	R = 0.14, b = 0.36	R = 0.15, b = 0.33		
		flex.	D		R = 0.1, b = −0.29		**R** = **0.53, b** = **0.86**	*R* = *0.27, b* = *0.51*	*R* = *0.24, b* = *0.41*		
			ND	R = 0.09, b = 0.47			*R* = *0.34, b* = *0.82*	R = 0.17, b = 0.45	*R* = *0.28, b* = *0.5*		
**Hand Grip Strength (Kg)**											
HGS			D				**R** = **0.56, b** = **0.01**	**R** = **0.39, b** = **0.01**	**R** = **0.29, b** = **0.01**		
			ND				**R** = **0.6, b** = **0.01**	**R** = **0.47, b** = **0.01**	**R** = **0.34, b** = **0.01**		
**Balance score (Z-score)**											
AP			EO					R = 0.13, b = −0.1			
			EC								
ML			EO								
			EC								
**Proprioception (deg)**											
max. error			D								
			ND								
min. error			D								
			ND								
mean error			D								
			ND								
s.d. error			D								
			ND								
**Joint Range of Motion (deg)**											
Hip	flex.		D				R = 0.13, b = 0.01				
			ND								
Ankle	pflex.		D					R = 0.15, b = −0.01			
			ND							R = 0.08, b = 0	
	dflex.		D				R = 0.12, b = −0.01				
			ND								
**Nerve Conduction Study**											
CMAP (mV)	median						R = 0.16, b = 0.02				
	tibial						*R* = *0.28, b* = *0.02*				
	peroneal						*R* = *0.25, b* = *0.04*		R = 0.12, b = 0.02		
PMCT(ms/cm)	median										
	tibial				R = 0.26, b = 9.26		R = 0.23, b = −11.79				
	peroneal										
Hlat(ms/cm)	tibial						R = 0.24, b = −4.6			R = 0.11, b = 1.7	

The adjusted *R*^2^ (*R*) and normalized slope of the linear regression (*b*) are provided if a significant regression was found (*p* < 0.05). *Italic* indicates regressions with *p* < 0.01, in **bold** regressions with ***p*** < **0.001**.

##### Capacity

VERVEL showed significant regressions with strength measures in SELF (Table 4). VERVEL had the strongest regression with hip extension strength (R^2 = 0.31–0.50) and HGS (dom: R^2 = 0.39, non-dom: R^2 = 0.47) followed by the measures for hip flexion (R^2 = 0.24–0.32), and knee (R^2 = 0.13–0.25), ankle (R^2 = 0.09–0.14), and elbow (R^2 = 0.13–0.27) isokinetic joint strength (Table 4). This indicates that stronger participants had a higher upwards COM velocity in standing up. In FAST, the regression between VERVEL and the strength measures were generally weaker than in SELF (Table 4).

HORVEL had strong significant (*p* < 0.001) positive regressions in FAST with the isokinetic joint strength of the knee (R^2 = 0.41–0.69), hip (R^2 = 0.41–0.64), ankle (R^2 = 0.21–0.56), elbow (R^2 = 0.34–0.54), and HGS (dom: R^2 = 0.56, non-dom: R^2 = 0.60), indicating that stronger participants had a higher peak HORVEL in standing up in FAST not in SELF. These findings were underlined by the positive regressions found with the CMAP amplitudes, and the regression results found for TSTEP (Table 4). HORVEL and TSTEP also showed significant negative regressions with PMCT and H-lat at the tibial site, indicating that participants with longer latencies moved slower in the horizontal direction in FAST. There were some small significant negative relationships in SELF between rTUL and knee extension, and hip flexion indicating that stronger participants had started unloading their swing foot earlier (Table 4). A positive relationship was found for H-reflex latencies, indicating that participants with longer latencies also had a later rTUL (*p* = 0.035).

##### Task demand

**Fig. 6:**
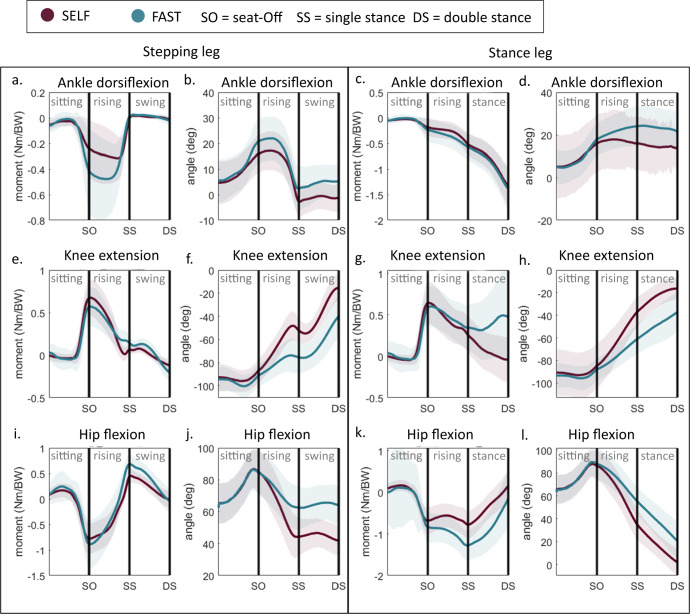
Joint moments and angles during sit-to-walk at distinct velocities. Trajectory comparison between FAST and SELF for the lower limb joint moments and angles.

The largest relative differences in increase of joint moments in FAST compared to SELF are the ankle plantarflexion moment of the stepping leg and the hip extension moment in the stance leg in the rising phase (Fig. 6). In the stance/swing phase, there is a significant increase in knee extension moment in the stance leg. The knee, hip, and ankle joints maintain more flexion during the rising and swing/stance phases in FAST, indicating that participants increased their HORVEL by stepping before their joints were fully extended.

## Discussion

This study identified compensatory strategies in the vital sit-to-walk movement and collected detailed neuromuscular capacity measures in a number of young and elderly adults. We assessed which age-related declining variables of capacity or altered movement objectives show trends of a relationship with compensatory behaviour. The elderly men used very similar consistent movement strategies in standing up, pushing-off with their arms. This links to the fundamental idea that humans select similar movement patterns that might be considered optimal for a specific task. This study emphasizes this by showing that as functional redundancy decreases due to ageing, similar adaptation strategies are used. With the detailed capacity measures we were able to link compensation to some of the age-related alterations in the neuromusculoskeletal systems, reinforcement schemes (movement objectives), and altered sensory inputs.

The participants in this experiment can be considered a relatively normative group of young and elderly adults as HGS is a commonly used estimate for overall muscular capacity and frailty and the mean measured HGS here is in agreement with the 50% percentile of normative HGS values for all four age-sex groups^9^.

### Arm strategies

It is regularly implied that adults use their arms to rise from a chair to compensate for muscle strength reduction. In this experiment, however, most elderly men used the armrests to stand up, but the elderly men were generally not weaker in the upper and lower extremities than the young women who did not use the armrests, even when corrected for body weight. These isokinetic strength measurement results are in line with previous reports^10^, and the CMAP results from the nerve conduction study confirm this finding. Using an armrest push-off resulted in a large reduction of the lumbar joint extension moment. Lumbar joint strength capacity was not captured in this study, however, previous studies report that elderly men also do not differ from young women for trunk strength^11^. We therefore conclude that it is not an absolute lack of or a reduction in strength that makes healthy elderly adults adopt their arm strategies in standing up; the results suggest that the use of arms is more likely related to the (perception) of stability.

ARM had lower maximal hip and ankle joint range of motion than LEG. Beissner et al. (2000)^12^ report findings that are in line with these results. After running a step-wise multivariate regression to determine the contributions of impairments to a Physical Performance Test, the main contributor to their regression was the lower-limb range of motion, followed by the lower limb strength. We hypothesize that as the ankle joint range of motion is most connected to the arm adaptation strategies, arm adaptation strategies might be related to postural controllability. Although only a biomechanical constraint, ankle ROM limitations limit the ability to use an ankle strategy or compensatory steps for postural recovery after perturbation^13^. So reduced ankle ROM is one of the biomechanical factors that increases fall risk. Our results show that all participants with an increased fear of falling, consistently used their arms to push-off in standing up, including one young man. This indicates that (perception of) stability probably is an important objective in arm strategy adaptation. Note that therefore not necessarily the absolute capacity is of importance but the relative reduction in capacity may lead to the perception of instability or lack of control. The relative muscular strength reduction is larger in men than in women which could relate to the more prominent use of armrest push-off by older men.

The absolute joint range of motion of participants during the STW tasks of all strategies would have been within the maximal joint range of motion of each participant. On average, ARM did have lower dorsiflexion ankle angles during movement compared to LEG. We therefore propose that humans start to compensate (reducing the task demand) prior to coming up against capacity limits, to maintain or increase their reserve, which is in line with humans’ sensory integration theories^1^. Bayesian interference is a mathematical framework used to model how the brain deals with uncertainty in the perceptual, motor, and cognitive domains^1^. The fundamental idea is that if we know the level of noise in our sensory system, based on a current sensory input the likelihood (or probability) of where e.g. one of our ligaments is in space can be estimated. This implicitly results in a cloud of uncertainty of the state estimate of our body in space. Additionally, there is an environmental uncertainty, both due to noise in the perceptual systems, and external events, such as sudden perturbations, and there are motor uncertainties due to the dynamics of the motor system. In this argumentation, it seems only reasonable that if functional redundancy reduces with age, humans will always prefer to keep a minimal threshold on neuromuscular reserve to cope with the many uncertainties and thus start to compensate ahead of hitting their physical boundaries.

Another argument of why the use of arms in standing up is balance-related rather than physical support, is that some participants put on a low force (<7 N) on the arms. Two reasons could then be considered for placing the arms on the armrest or on the thighs. First, as an emergency fall-back option in case of unexpected disturbances. Second, sensory motor research of the last two decades shows that a light touch contact, like a tip of one finger with a stable environmental surface (1 N), can significantly reduce the postural sway in standing balance^14^. The additional force and position information from the armrests or thighs thus adds to the sensory information and might improve the balance control in standing up.

Joint range of motion, and specifically ankle range of motion, could be a significant early clinical indicator of functional decline. Whether the ankle range of motion reduction indeed is a cause, or whether it is a result of adaptation requires further study. If it is a cause, flexibility training, like Tai Chi^15^ or Yoga, could lead to delayed adaption and possibly reduced fall risk.

### Foot positioning

In sit-to-walk we found that weaker adults (lower joint strength, CMAP amplitudes, and HGS) and adults with longer nerve conduction latencies used a smaller base-of-support while rising from the chair. This is contrary to what was expected. Gait studies have observed that elderly adults, compared to young adults in their 20 s, walk with a wider base-of-support^16,17^. Moreover, intuitively someone would widen their stance when situations become more challenging. A wider base of support allows for larger centre-of-mass excursions due to e.g. external perturbations before a corrective step is necessary.

Since strength measures were significantly different between sexes, we first considered that the smaller BOS could be a cultural and societal artefact. Originating from cultural customs, women are implicitly supposed to sit with their feet closer together than men. However, we found similar regressions and trends if only the men were considered. Even within each age-sex group, although the groups are then small, the trend is similar for most strength measures. Participants with a wide BOS showed significantly higher hip and knee extension moments in the rising phase for the stepping leg compared to participants that used a medium or a wide range of support. Possibly, placing the feet wider apart requires larger knee and hip extension moments in the stepping leg or alternative muscle recruitment loading muscles that are more prone to age-related regression; however, this cannot indisputably be concluded from this data collection, since each participant used alternative arm strategies and anterior-posterior foot positions^18^. It requires a separate experiment or biomechanical simulations to test this hypothesis^19^. If the lower joint moments are a result of the lower joint strength capacity, this shows that participants adapt their strategies ahead of the physical boundaries, linking to the sensory uncertainty theory.

A smaller base of support generally results in a smaller mechanical stability margin. At the same time, many of the falls in the elderly occur during standing up. If a reduced lower limb strength is the cause of a smaller BOS, strength training not only helps in recovery after perturbation, but would also enable a mechanical larger stability base in standing up.

Alternatively, we have to consider the neuromechanical interactions as proposed by Bingham et al. (2011)^20^ for standing balance. A wider stance in standing balance improves the stability in the presence of a perturbation due to the higher mechanical leverage. This allows for greater torque generation about the centre of mass with lower muscular efforts. The reduced torque required for stability at wider stance widths requires appropriately scaled delayed neural feedback. So, a wide base of support limits the set of feasible stable feedback gains. This theory might also translate to the BOS in STW; the results showed that participants with longer nerve conduction latencies had a smaller BOS. People with an increased sensorimotor delay probably have smaller feasible feedback gains for maintaining stability at wide stances. We therefore hypothesize that the negative relationship between the width of BOS and nerve conduction latencies is a compensation to reduce the neural demand for the task.

### Why do elderly adults stand up differently to young adults?

This study was able to link compensation in STW to some of the age-related alterations in the neuromusculoskeletal systems and reinforcement schemes. Ankle joint range of motion, strength, neural latencies, and the perception of, or emphasis on, stability play leading roles. The results also show that upward, but not forward movement speed was not related to capacity during the first phases of STW (sitting, rising, stepping) in SELF. When movement speed became a movement objective as tested in FAST, adults with higher strength measures and CMAP amplitudes did have a higher forward velocity. Pain was monitored with a VAS scale but was marginal and not related to compensation strategies in this experiment. Compensational strategies due to past trauma (pain) could only be analysed based on the self-reported medical history in the questionnaires; we hypothesize this to be of importance but could not investigate this with the data collected. The energetics objective was not quantified in this experiment; however, based on the results it is safe to assume that energetics is not the main movement objective in sit-to-walk.

The question whether compensation is a result of altered sensory inputs could not be fully answered within this experimental set-up. The sensory systems involved in postural control are the visual, auditory, vestibular, and somatosensory, including proprioception, exteroception (e.g. touch, pain), and interoception (e.g. respiration, cardiovascular system). In the current experiment, joint sense acuity of the knee as indicator of the status of the somatosensory system was analysed in an ipsilateral matching task of the knee joint position based on Hurley et al. (1998)^21^. The mean error value results were higher than measured in Hurley et al. (1998), but within range with other published sources^22^. However, contrary to literature, acuity of the elderly in this experiment did not significantly differ from the young. Individual datapoints did indicate that the chosen metric is indicative of the proprioceptive capacity, since three participants suffering from, respectively diabetes, osteoarthritis, history with knee infection, and/or knee replacement did have notable lower acuities than the other participants. We therefore have to consider that our relatively healthy group of elderly adults did not suffer from low knee joint sense acuity. Since the elderly in this study did show compensatory strategies in foot positioning and arm strategies, we consider that knee joint sense acuity is not the main contributor of early adaptive movement behaviour in elderly adults in STW.

Apart from the status of the auditory system, which was monitored in the questionnaire, other parts of the sensory system were not monitored in this study. Since the perception of stability seems to be an important driver, the status of the vestibular system, or the altered weighing of sensory inputs, might be important for how humans select their movement patterns. Moreover, we have to consider that the sensorimotor control is subject to motor and sensor noise. Motor and sensor noise interfere with control and are known to increase with age leading to adaptations^23^. This is why the neuromechanical interaction is important to consider^20^.

In conclusion, this study has shown that the interrelationship between stability, joint range of motion and strength is key to adaptations in the sit-to-walk activity, a vital determinant of mobility and a signature task of ageing. When functional redundancy reduces with age, humans prefer to maintain a minimal threshold of neuromuscular reserve to cope with the sensorimotor uncertainties. Therefore, even with similar reinforcement schemes, elderly adults will reveal early adaptation strategies, prior to coming up against physical limitations. Future work should address further associated questions, including the perception of stability, and the status of the vestibular system and noise in the sensorimotor control in relation to daily life adaptations. Biomechanical simulations of human movement should consider alternative simulations and cost functions to solve for functional redundancy, e.g. stochastic control.

#### Clinical relevance

One-third of the people above 65-years of age experience a fall annually and this is expected to increase in the upcoming years^24^. Elderly adults who have experienced a fall suffer physical and mental health problems like injuries, long-term institutional care^25^, fear of falling^26^, reduced activity^27,28^, and a lower quality of life. Medical fall-related costs are rocketing. Prevention via targeted intervention is key, which requires early identification of compensatory strategies and underlying physical or mental considerations. This article shows that joint range of motion, and specifically ankle range of motion, could be a significant early clinical indicator of functional decline. Maintaining and increasing flexibility in this group could be beneficial but requires further investigation. Lower limb strength training might empower elderly adults to use a wider base-of-support in standing up, which could reduce the risk of falls, however further study on the neuromechanical interaction is required.

#### Study limitations

- Due to the extensive experimental set-up, this study was limited to 50 participants.
- The perception of the unconventional instrumented chair might have an effect on the outcomes. To test the resulting hypotheses of this study, further studies could aim for experiments within the daily life environment.
- Apart from the status of the auditory system, other parts of the sensory system and noise in the neural system were not monitored within this study.
- The interpretation of the chosen balance assessment task is under debate; alternative tests of stability (and fall risk) could be considered, e.g. history of falls.
- Within the proprioceptive task, participants moved to a certain position until instructed to stop. Participants might have used the timing of this initial movement for position reconstruction instead of solely the proprioceptive feedback.

## Method

### Participants

This study comprises 27 young (Y) (20–35 years, 14 women) and 23 relatively healthy elderly (E) adults (65–95 years, 12 women) (total: N = 50) recruited between July and November 2019 in London, UK (Table 1). Participants were categorised into four age-sex groups: young women (YW), young men (YM), elderly women (EW), and elderly men (EM). Participants were excluded in case of: any history of severe mobility-limiting pathologies, any known allergies to adhesives, unable to speak or read English at a sufficient level to give informed consent, suffering from a neurological pathology, psychiatric illness or mental state that limits informed consent, any systemic inflammatory, connective tissue disorders or medical disorders that limit exercise, pregnancy, a pacemaker, neurological injury, or when identified as “Moderately” or “Severely frail” on the Edmonton Frailty Scale^29^. Participants visited the laboratory once for 3–4 h. In between tests participants were allowed to take as much rest as needed in a separate room with refreshments. Tests were run in parallel, starting either with the sit-to-walk tasks, or the capacity measures. This study received ethical approval by the institutional ethics committee of Imperial College London, UK. All participants gave written informed consent.

### Participant characteristics

A questionnaire assessed (former) profession, levels of activity, diet, general health, current or prior injuries, and level of frailty (Edmonton Frailty Score). Hand dominance was determined with the Edinburgh Handedness questionnaire^30^. Based on the information from the questionnaires, participants were marked in lifestyle groups of exercise frequency (never, 1–2 times/month, 1–2 times/week, >= 3 times per week) and the physical occupational demands (not physically demanding, 1–3 hr/day, 3–5 hr/day, >6 h per day);

### Sit-to-walk task

#### Instructions

Prior to instrumentation, participants performed a timed-up-and-go (TUG) test from a normal chair without armrests^4^. At the investigator’s signal, participants had to stand up, walk 3 m towards a cone, go around it, and return to their seat as fast as they could comfortably achieve this. Participants were timed and recorded on camera.

**Fig. 7:**
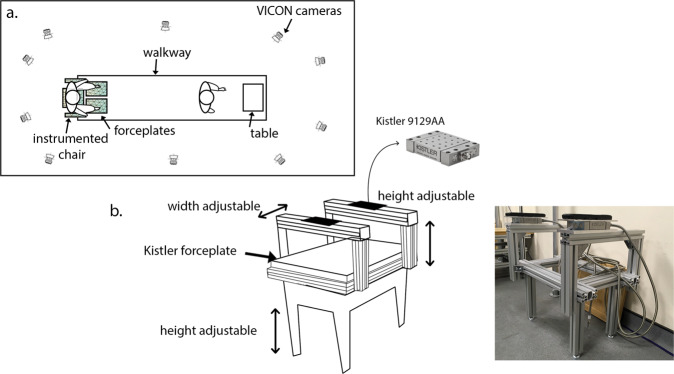
Experimental set-up. Participants sat down on an instrumented chair (**b**), with 6D force plates in the seat and in the armrests, and two force plates at the feet. 3 m in front of the chair was a table (**a**). Kinematics were captured with a Vicon camera system and reflective markers. Participants were further equipped with 16 EMG sensors.

After instrumentation, participants sat down on an instrumented chair with the seat adjusted to approximately knee height (mean = 46.7 cm, SD = 3.9 cm) and with instrumented armrests (Fig. 7). There were two sequential conditions, each repeated 5 times.

##### Self-selected speed (SELF)

participants started sitting down, then were instructed to pick up an object from a Table 3 metres in front of them (similar to Dolecka (2015) and Komaris (2018)^31,32^), and then returned to their seat (sit-to-walk (STW)). Participants were instructed to perform the task in a natural manner similar to standing up from a chair at home to pick up a phone from the table in front of them. In order to address the lack of compensation permitted in previous studies^5^, participants were not given any instructions on how to move. If a participant asked for instructions, the investigator’s reply was ‘to move as you normally would in your own home’.

##### Fast speed (FAST)

in this consecutive condition there was a timer at the table. Participants were instructed to reach the table as fast as possible, stop the timer and then return to their seat. Participants were verbally encouraged to go as fast as possible.

#### Motion capture

Participants were equipped with 16 EMG sensors and 84 reflective markers (Supplementary Tables 1, 2). The 14 mm-diameter spherical markers were attached to the thorax, arms, pelvis, legs and feet using double-sided tape, either separately or as clusters of four. Reaction forces were measured via two Kistler forceplates embedded in the walkway and one at the seat, and two 9129 AA Kistler forceplates in the armrests (Fig. 7). A Vicon system with 10 cameras (MX T20) captured the STW volume (100 Hz). A wireless Delsys EMG system with 16 sensors measured the EMG signal of specific muscle groups. The European guidelines for the EMG sensor placement were followed^33^. Marker trajectory, force, and EMG data were exported to Matlab R2018b for inverse kinematics and analysis.

#### Categorization movement strategies

In literature, movement strategies in sit-to-stand had been divided into momentum transfer, exaggerated trunk flexion, and dominant vertical rise^5^. This division was mostly based on the synchronisation between trunk flexion, knee extension and forward-upward centre of mass (COM) velocity, and applied in experiments where the use of arms was restricted. These definitions are not suitable for sit-to-walk strategies as in this study participants usually stepped before extending their trunk (lumbar extension) and velocities of the COM were higher since the COM does not have to come to a stop; additionally, the initial COM-base of support (BOS) distance was freely chosen. We therefore categorized the observed strategies based on the use of arms and foot positioning. Movement strategies were analysed at the frame just before the participant left the seat (Seat-Off (SO)) determined based on the reaction forces measured by the forceplate on the seat:

- *Arm strategies* were classified as armrest push-off, thigh push-off, swinging the arms, and not using the arms. Strategies were categorised using a cluster technique following Komaris et al. (2017)^32^ and then manually checked using the Vicon Nexus v2.9.1 software. A strategy was labelled as asymmetric if the participant used different strategies between left and right, e.g. when only one arm pushed off on the armrest, while the other was in swing.
- For the *foot positioning* the ankle joint centre and the midpoint between the toe markers were used to determine the medial-lateral distance (ML-BOS) (i.e. width of the BOS), and the anterior-posterior distance (AP-BOS) (asymmetry in foot positioning) at seat-off. To enable comparisons, ML-BOS was divided by the pelvis width (cML-BOS) and AP-BOS was divided by leg length (cAP-BOS).
- For pacing, the time between the initiation of the movement (T0) (based on the head movement in the sagittal plane) and when the foot that start in swing touches the ground (end of first step) (TSTEP) (determined by the forceplates on the floor) was used as measure of movement time. The trajectories of the COM velocity (VEL) and acceleration (ACC) in vertical (VERVEL, VERACC) and horizontal (HORVEL, HORACC) directions were compared between groups. Initiation of unloading of the swing foot relative to the peak vertical velocity was determined, further referred to as *rTUL*(previously described as ‘phase III unloading’^34^). A negative value indicates that the foot was unloaded prior to the peak COM vertical velocity.

#### Inverse dynamics

Participants were modelled as a chain of 15 linked rigid bodies, or segments: feet, legs, thighs, pelvis, HT (head & torso), upper arms, lower arms, hands. The global reference frame xyz is specified, where y is up, x is in the longitudinal direction and z is in the lateral direction, in agreement with the International Society of Biomechanics convention. The Euler rotations of a segment correspond to the order Y, X and Z. The joint rotation, which is the rotation between two segments, is rotated in the Euler sequence Z, X, and Y, around the segment coordinate system of the proximal segment, further referred to as the flexion-extension (Z′), internal-external rotation (Y″) and adduction-abduction (X‴). For each segment, the Newton-Euler equations of motion were determined in the global reference frame^35^. The centre of mass (COM) and the mass and inertial tensor specifications of the separate segments were determined by the specifications given in Zatsiorksy and de Leva (1996) for the young subjects^36,37^ and in Pavol et al. (2002) for the elderly participants^38^. External forces were measured with the force plates on the floor, seat, and armrests. Note that no inverse dynamics analysis could be conducted for participants that pushed off on their thighs, as these contact forces were not measured during STW.

### Capacity measures

#### Isokinetic strength measures

Participants’ maximum isokinetic strength was measured with the Cybex Humac CSMI dynamometer for the dominant and non-dominant side. The measurements were done for the hip, knee, ankle, and elbow for flexion and extension at two angular velocities: 60 deg/s and 90 deg/s. Joints were carefully aligned with the axis of the apparatus. Hip flexion and extension was measured in supine position from 90° flexion to 0° and back to 90° flexion with the contralateral leg with 0° hip flexion and 90° knee flexion. Knee flexion and extension was measured in sitting position from 90° flexion to 5°, to prevent overextension. Ankle dorsi- and plantarflexion was measured in supine position from 5° dorsiflexion to 5° plantarflexion with the ipsilateral hip and knee slightly bent. The elbow joint was measured from 0° to 90° flexion in supine position with the hip fully extended and the knees flexed at 90°. Participants were verbally encouraged to push as hard as possible during the trials. For each new condition, participants did two test trials followed by three repetitions for the actual measurement. The maximum peak isokinetic joint moment of the three repetitions was determined for each condition. All measures were normalised to bodyweight (BW).

#### Handgrip strength (HGS)

Low hand grip strength (HGS) has been shown to predict disability, hospitalization, and mortality^39^. HGS was therefore included and measured with a Jamar hand-held dynamometer both on the dominant and the non-dominant hand. Participants were sitting down and were encouraged to squeeze the dynamometer as hard as possible. Three trials were conducted on each side; the highest value was taken as the maximum handgrip strength (max HGS).

#### Balance

To assess balance, participants were instructed to stand as still as possible for 30 seconds or until loss of balance (i.e. stepping out) while standing on a force plate. Their feet were placed such that the medial malleoli were close together, and their arms were crossed over their chest to avoid arm sway. The experiment was done with eyes open (O) and eyes closed (C).

Reconstructions of the centre of pressure (CoP) movement in the anterior-posterior (AP) and medial-lateral (ML) direction were determined from the force plate data. The mean CoP position was subtracted from the measured CoP positions to obtain relative measures of mean amplitude, amplitude variability, mean velocity, velocity variability, and range. Based on Pasma et al. (2014), each of these parameters was then transformed into a z-score resulting in standardized CoP parameters with a mean of 0 and a standard deviation of 1. By averaging the z-scores, a composite score was constructed for two directions (AP,ML) in both conditions (O,C). The composite score combines the single CoP parameters to have a more consistent measure^40^.

#### Proprioception (PROP)

To assess proprioceptive acuity, participants performed an ipsilateral matching task of the knee joint position based on Hurley et al. (1998)^21^. In a quiet environment, participants were blindfolded and seated on the edge of a bench with their hips and knees flexed at approximately 90° and their lower leg hanging freely. Participants were instructed to slowly straighten their knee until the investigator told them to stop. The knee was now flexed at an angle between 0 and 90°: the ‘test angle’. For approximately 5 s participants were asked to visualize their current knee position. Then participants were instructed to relax, allowing their leg to hang freely in the resting position. After 3 s the participant was asked to reproduce the test angle: the “reproduced angle”. This procedure was repeated for 10 trials for each leg. Knee flexion was estimated with the Vicon Motion Capture system. Angles were chosen randomly by the researcher throughout the range of 90° flexion to full knee extension. The minimum, maximum, mean and standard deviation of the error of the 10 trials were calculated and used as an indication of the proprioceptive acuity (knee joint position sense).

#### Joint range of motion (JROM)

The joint range of motion of the hip and ankle on the Cybex Humac CSMI dynamometer were assessed. For the hip, participants were supine and asked to pull their leg towards their chest, with the knee flexed at 90°. The contralateral leg had a fully extended hip and an approximate knee flexion of 90°. For the ankle, participants were in supine position, with a small flexion in the ipsilateral knee and hip, the contralateral hip fully extended, and the contralateral knee at approximately 90°. The investigator moved the ankle to the outer positions until the participant indicated that they reached their maximum joint position.

#### Nerve conduction study (NCS)

A nerve conduction study (NCS) assessed participants’ neural capacity^41^. During NCS brief electrical stimuli are delivered to a nerve to determine how fast the nerves are conducting an electrical current and the maximum excitation of the muscle. The Median (at the wrist), Tibial (at the popliteal fossa) and Peroneal (at the fibula head) nerves were stimulated bilaterally using a constant current stimulator (DS7, Digitimer, Welwyn Garden city, UK) that generates brief square-wave pulses. Electromyographic (EMG) activity was recorded using pairs of self-adhesive electrodes (Ag/AgCl, Kendall, Henleys Medical Supplies, UK) positioned on the skin overlying Abductor Pollicis Brevis (ABP), Soleus, and Extensor Digitorum Brevis muscles. The electrodes were positioned parallel to the muscle fibre orientation. A ground electrode was placed in the palm of the hand for the APB and over the left lateral malleolus for the other sites. EMG data were filtered (10–1000 Hz), amplified (1000×; Iso-DAM, World Precision Instruments, UK) and sampled at 2 kHz using a Power 1401 data acquisition system and Signal v5 software (Cambridge Electronic Design [CED], UK) connected to a computer for subsequent offline analysis in Matlab. Stimulation was slowly increased from 0 mA in steps of 0.5 mA. At low intensity, stimulation preferentially activated sensory fibres and triggered an H-reflex, in which the signal travels via the sensory nerve to the spinal cord triggering a reflex that involves activation of the muscle via the motor axons of the nerve. The latency between the stimulus and the muscle activation is the H-reflex latency (Hlat). The Hlat was only determined for the Tibial Nerve stimulation on both sides. By increasing the intensity of the stimulation, the muscle was activated directly by the applied stimulus travelling along the motor nerve towards the muscle. The size of this wave, the compound motor action potential (CMAP) is proportional to number of muscle fibres that are depolarized. The peak-to-peak amplitude CMAP is reported. Five maximal motor responses were recorded at the same intensity. An intensity of 120% of the intensity used to elicit the maximum CMAP was delivered at 1 Hz until 10 F-waves were recorded. An F-wave is triggered when the stimulus travels antidromically in the motor nerve towards the spinal cord and then returns orthodromically down the motor nerve to the muscle. The F-wave latency is the time between the stimulus and the muscle response. The peripheral motor conduction time (PMCT) was calculated by PMCT = (CMAP latency + minimum F-wave latency-1)/2. PMCT eliminates differences in stimulating electrode positioning between participants. A 1 ms delay was subtracted to account for the action potentials to turn around in the motor neuron cell body in the spinal cord. The corrected measures cHlat and cPMCT are respectively the H-latency and peripheral motor conduction time divided by the participants’ height.

### Movement objectives

#### Stability: fear of falling

Adults may put more emphasis on stability during movement if there is an increased fear of falling. A questionnaire was used to assess fear of falling (FES-I short^42^). This 7-item questionnaire produces a score that is related to their fear of falling: score 7–10 – hardly afraid to fall; score 11–28 very afraid to fall. Also, loss of hearing and level of dizziness were assessed in the questionnaire, as they have been associated with movement alterations^43^.

#### Pain avoidance

Pain avoidance is a contributor for compensation for movement objectives. At the start and the end of the experiment, participants were asked if they were currently experiencing any levels of pain (including muscle strain) on a visual analogue scale (VAS) from 0 (no pain) -10 (maximum pain)^44^.

### Statistics

Differences in means between age-sex groups, strategy groups, and lifestyle groups were assessed with a one-way ANOVA and a pairwise comparison between groups to determine significant differences (MATLAB). The relationship between BOS and capacity measures was determined using a linear regression analysis, and if further analysis was required, a stepwise multivariate regression. For trajectory comparisons of the joint moment, joint angles, COM velocity and acceleration, and external forces, two-sided *t* tests were performed for every time sample. The level of significance was visualized as the negative base-10 logarithm of the *p* value so that large values represent small *p* values, similar to^45^. Note that despite the relatively conservative corrections of the significance level, these comparison metrics should be indicative.

## Supplementary information

Supplemental material

## Data Availability

Data on the statistical analyses are available via the opensource visualisation application online (http://bodieslab.com/data/). Additional datasets generated and/or analysed during the current study are available from the corresponding author on reasonable request.

## Code availability

Code on the statistical analyses are available via the opensource visualisation application online (http://bodieslab.com/data/). Additional code on the kinematic modelling are available from the corresponding author on reasonable request.
