# Supplementary material for: Why do older adults stand-up differently to young adults?: investigation of compensatory movement strategies in sit-to-walk
Source: NPJ Aging. 2022 Sep 5;8(1):13. doi: 10.1038/s41514-022-00094-x (PMC9445096; doi:10.1038/s41514-022-00094-x)
Supplement: Supplementary file 1 — Supplemental material [file 41514_2022_94_MOESM1_ESM.pdf]

Supplementary Table 1 - Anatomical locations of reflective markers. Total number of markers = 84

Segment	Markers	Nr.	Anatomical location
Thorax	C7	1	7th cervical vertebra
	T8	2	8th thoracic vertebra
	IJ	3	Sternum jugular notch
	MA	4	Sternum manubrium
	PX	5	Sternum xiphoid process
Pelvis	RASIS	6	Right anterior superior iliac spine
	LASIS	7	Left anterior superior iliac spine
	RPSIS	8	Right posterior superior iliac spine
	LPSIS	9	Left posterior superior iliac spine
	P1,P2,P3	10	Cluster of three markers placed on the pelvis
Clavicle	LA	11	Left Acromioclavicular joint
	RA	12	Right Acromioclavicular joint
	SC	13	Sternoclavicular joint
Scapula	S1,S2,S3	14	Scapula trackers (3 markers) placed on both scapula spines
Humerus	HU1,HU2,HU3,HU4	15	Cluster of four markers placed on the upper arm
	LE	16	Lateral epicondyle
	ME	17	Medial epicondyle
Forearm	U1,U2,U3,U4	18	Cluster of four markers placed on the forearm
	US	19	Ulnar styloid
	RS	20	Radial styloid
Hand	H1,H2,H3	21	Cluster of three markers placed on the back of the hand
Foot	FM2	22	Head of the second metatarsal
	FCC	23	Calcaneus
	FMT	24	Tuberosity of the fifth metatarsal
	TF	25	Additional marker placed on the foot
	FAM	26	Apex of the lateral malleolus
	TAM	27	Apex of the medial malleolus
Shank	C1,C2,C3,C4	28	Cluster of four markers placed on the calf segment
Knee	FLE	29	Lateral femoral epicondyle
	FME	30	Medial femoral epicondyle
Thigh	T1,T2,T3, T4	31	Cluster of four markers placed on the thigh segment

Supplementary Table 2 - EMG sensor placement

Sensor nr.	Abb.	Muscle
Left		
1	SOL_L	Left Soleus
2	GAS_L	Left Medial Gastrocnemius
3	TA_L	Left Tibialis anterior
4	HAM_L	Left Biceps femoris long head
9	VAS_L	Left Vastus Lateralis
10	GLU_L	Left Gluteus maximus
11	RF_L	Left Rectus Femoris
12	HAB_L	Left Gluteus Medius
Right		
5	SOL_R	Right Soleus
6	GAS_R	Right Medial Gastrocnemius
7	TA_R	Right Tibialis anterior
8	HAM_R	Right Biceps femoris long head
13	VAS_R	Right Vastus Lateralis
14	GLU_R	Right Gluteus maximus
15	RF_R	Right Rectus Femoris
16	HAB_R	Right Gluteus Medius

Supplementary Table 3 To analyse task demand in relation to BOS, a trajectory comparison was done in which participants were divided into three BOS categories (wide, medium, small), based on their cML-BOS during rising. The Peak significant differences in joint moments, obtained via the trajectory analysis, are provided.

Foot strategy groups		Wide	Medium	Small
SELF				
Group		N = 14	N = 14	N = 16
		5 YW, 7EW, 2EM<br>---------------------<br>5 ARM, 1 THIGH, 3 LEG, 5 OTHER	5 YW, 4YM, 4EW, 7EM<br>-----------------------<br>6 ARM, 2 THIGH, 5 LEG, 4 ARMF, 5 OTHER	4 YW, 9YM, 1EW, 2EM<br>--------------------<br>4 ARM, 1 THIGH, 5 LEG, 1 ARMF, 5 OTHER
**Joint moments**	**Timing (rise phase)**	**(Nm/BW)**	**(Nm/BW)**	**(Nm/BW)**
Hip Extension (step)	55%	-0.62		-0.39
	25%	-0.82	-0.61	
Knee Extension (step)	5%	0.77		0.65
	17%	0.74	0.56	
FAST				
Group		N = 14	N = 25	N = 11
		4 YW, 8EW, 2EM	8 YW, 6YM, 4EW, 7EM	2 YW, 7YM, 2EM
		4 ARM, 1 THIGH, 3 LEG, 6 OTHER	9 ARM, 1 THIGH, 7 LEG, 5 ARMF, 3 OTHER	3 ARM, 2 THIGH, 3 LEG, 4 OTHER
Joint moments	timing	(Nm/BW)	(Nm/BW)	(Nm/BW)
Hip extension (step)	-4%	-1.14	-0.72	
	40%	-0.84		-0.48
Knee extension (step)	14%	0.73	0.48	
	9%	0.74		0.54
